# Protocol for a randomized, double blind, placebo controlled, crossover trial of Melatonin for treatment of Nocturia in adults with Multiple Sclerosis (MeNiMS)

**DOI:** 10.1186/s12883-017-0845-y

**Published:** 2017-03-27

**Authors:** D. Delgado, L. Canham, N. Cotterill, D. Cottrell, M. J. Drake, K. Inglis, D. Owen, P. White

**Affiliations:** 1 Bristol Urological Institute, North Bristol NHS Trust, Southmead Hospital, Bristol, BS10 5NB UK; 2 Department of Neurology, North Bristol NHS Trust, Southmead Hospital, Bristol, UK; 3 School of Clinical Sciences, University of Bristol, Bristol, UK; 4 University of West of England, Bristol, UK

**Keywords:** Multiple Sclerosis, Nocturia, Melatonin, LUTS, Cognition

## Abstract

**Background:**

Nocturia (the symptom of needing to wake up to pass urine) is common in progressive Multiple Sclerosis (MS) patients. Moderate-to-severe nocturia affects quality of life, can exacerbate fatigue and may affect capacity to carry out daily activities.

Melatonin is a natural hormone regulating circadian cycles, released by the pineal gland at night-time, and secretion is impaired in MS. Melatonin levels can be supplemented by administration in tablet form at bedtime.

The aim of this study is to evaluate the effect of melatonin on mean number of nocturia episodes per night in MS patients. Secondary outcome measures will assess impact upon quality of life, urinated volumes, lower urinary tract symptoms (LUTS), cognition, sleep quality and sleep disturbance of partners.

**Methods:**

A randomized, double blind, placebo controlled, crossover trial consisting of two, six week treatment phases (active drug melatonin 2 mg or placebo), with a 1 month wash-out period in between.

The primary outcome (change in nocturia episodes per night) in this two arm, two treatment, two period crossover design, will be objectively measured using frequency volume charts (FVC) at baseline and following both treatment phases. Questionnaires will be used to assess quality of life, sleep quality, safety and urinary tract symptoms. Qualitative interviews of participants and partners will explore issues including quality of life, mechanisms of sleep disturbance and impact of nocturia on partners.

**Discussion:**

This study will evaluate whether melatonin reduces the frequency of nocturia episodes in MS patients, and therefore whether ‘Circadin’ has the potential to reduce LUTS and fatigue, and improve cognition and overall quality of life.

**Trial registration:**

(EudraCT reference) 2012–00418321 registered: 25/01/13. ISRCTN Registry: ISRCTN38687869

## Background

Normal urinary function shows a circadian (day/night) cycle, with reduced urine production and bladder excitability during sleep. Nocturia is the symptom of needing to wake up to pass urine. It is caused by increased bladder excitability, impaired salt/fluid handling and endocrine causes.

Nocturia is common; overall prevalence of nocturia is substantial, and progresses in severity with ageing [1]. Nocturia is highly prevalent in neurological disease. Mild nocturia (1 episode per night) may be regarded as a “nuisance” symptom, of minor importance. However, if there are other reasons for sleep disturbance, as is common in multiple sclerosis (MS) [2], the additional episode of sleep disturbance from nocturia becomes more important for the person affected.

Moderate-to-severe nocturia profoundly affects quality of life as a consequence of fatigue, which results from the shortening of overall sleep duration and disruption of the phases of sleep (loss of the restorative phase). More importantly, severe nocturia is associated with major health problems [3]. The condition is thus important personally (impaired quality of life from fatigue), economically (impaired function affecting employment; for disabled or elderly people, need for additional care and reduced ability to maintain independent living at home) and clinically (association with major health issues).

Standard treatment comprises controlling fluid intake, timed diuretics, desmopressin, antimuscarinic drugs or bedtime sedatives/miscellaneous compounds [4]. Nocturia can be multifactorial, and a range of interventions is needed to individualise treatment selection and have the best chance of ameliorating symptoms. Current options can have side effects- which are common with antimuscarinics (dry mouth, constipation, swallowing difficulty, cognitive impairment), and sedatives (confusion, sedation hangover). Serious complications include; hyponatraemia (salt depletion/dilution) with diuretics/desmopressin, and acute retention of urine with antimuscarinics. Perceived poor efficacy and the safety concerns mean that clinicians may fail to evaluate fully or feel reluctant to instigate treatment; as a consequence, even severe nocturia symptoms may be left untreated.

Our conjecture is that nocturia signifies impaired circadian regulation, potentially mediated at 3 levels; (i) bladder muscle excitability, (ii) renal urine output, (iii) conscious response to bladder sensation. The concept that nocturia represents a disruption of circadian control of various body processes (bladder urine storage, kidney urine production and brain response to urinary sensation) is evident. It shifts emphasis from organs to the individual person; thereby the treatment basis moves from suppressing organ activity (antimuscarinics, antidiuretics, sedatives), towards restoration of deficient circadian activity. This shift takes a holistic approach aiming to restore normal function in the organism, rather than suppressing abnormal function in individual organs; improved function by restoration of the native hormone. Melatonin is a natural hormone regulating circadian cycles, released by the brain’s pineal gland at night-time, and varying with sleep state. It reduces smooth muscle activity including in the bladder [5]. Melatonin levels can be supplemented by administration in tablet form [6]; given at bedtime, it improves sleep quality. Literature review shows melatonin has been used for nocturia in two small studies. In men with benign prostate enlargements, a subgroup of patients who benefitted from exogenous melatonin treatment (“nocturia responders”) has been reported [7]. Melatonin has also been used for nocturia in the elderly [6], for whom it was found to have significant benefit in ameliorating symptom severity and improving quality of life.

MS is the commonest cause of acquired neurological disability in young adults and is characterised by the progressive inflammatory loss of myelin and neuronal tissue in the central nervous system. In both the initial Relapsing-Remitting Phase and subsequent Progressive Phase of MS urinary symptoms are common, including nocturia, along with sleep disturbance from other causes (e.g. restless legs syndrome, anxiety). The impact upon quality of life is substantial; fatigue leads to reduced capacity to carry out daily activities, which is a particular problem in a disabled patient group. Fatigue itself is the most common symptom of Multiple Sclerosis and the main disabling feature for many patients. There is an absolute paucity of effective therapies for this severe symptom which is considered to be multifactorial and greatly exacerbated by all forms of sleep deprivation and disturbance. Fatigue is well recognised as a key determinant in a patient’s capacity to maintain occupation, independence and social participation.

Impaired melatonin secretion occurs in multiple sclerosis [8, 9]. Melatonin may have anti-inflammatory and neuroprotective effects [10–12], which are potentially relevant when considering its use in MS. We propose that exogenous administration of melatonin may counteract symptom severity in nocturia, with lower risk of adverse effects than current available agents. Accordingly, the main hypothesis is that bedtime administration of a 2 mg melatonin sustained-release tablet will improve clinical nocturia with consequent benefit for sleep quality and quality of life, in patients with progressive MS.

## Study aims

The primary aim is to evaluate effect of melatonin on mean number of nocturia episodes per night.

Secondary aims are to evaluate; 1) Improvement in quality of life, 2) safety, 3) lower urinary tract symptoms (LUTS), 4) total voided (urinated) volume and mean volume per void, 5) sleep quality and 6) sleep disturbance of partners. A sub-group of volunteers will also participate in assessments of Cognition.

## Methods

### Study design

This trial is a double-blind randomised placebo controlled crossover clinical trial to compare sustained-release melatonin (‘Circadin’ 2 mg) against placebo. A run-in phase will be followed by two treatment phases (active drug or placebo) of 6 weeks each separated by a wash-out interval of 1 month.

The treatment phases will be 2 mg per night (taken at bedtime) of capsulated sustained-release melatonin (‘Circadin’) or 1 placebo capsule per night, identical in appearance to ‘Circadin’; order of administration will be double-blind randomized.

Return to baseline after washout between the two treatment phases will be assessed with the International Consultation on Incontinence-Nocturia (ICIQ-N) and Cambridge Multiple Sclerosis Basic Score (CaMBS).

### Study setting and participants

This trial will recruit multiple sclerosis patients from North Bristol NHS Trust. Eligible subjects must be over 18 years of age with a confirmed neurological diagnosis of MS and least 1 episode of nocturia (as defined by International Continence Society criteria [13]) every night. In addition female subjects of child-bearing potential must agree to use an effective method of contraception throughout the study.

Subjects will be excluded if they i) are pregnant, ii) have a symptomatic urinary infection or iii) indwelling catheter, iv) use prescription/non-prescription sleeping tablets or Melatonin, v) use desmopressin or investigational medical compounds in the month preceding randomization vi) take antimuscarinic or diuretic medication, unless used long-term prior to study (at least 3 months) and continued at same dosing regime throughout the study, vii) have Diabetes mellitus or Diabetes insipidus.

All subjects will provide informed written consent prior to involvement in the study. The NRES Committee South West – Exeter have approved the study protocol (REC reference number: 12/SW/0322). A request for a clinical trial authorisation was accepted by the UK MHRA (reference; 18524/0222/001-0001). The study is registered in the BioMed Central ISRCTN Registry (http://www.isrctn.com/ISRCTN38687869).

Any protocol modifications that become necessary during the course of the trial will be discussed with all relevant parties in advance.

### Planned interventions

Baseline clinical assessment

- Neurological diagnosis and medical history
- Medication review
- Dipstick urinalysis to screen for urinary tract infection (specifically evaluating for presence of nitrites)
- Nocturia assessment tool (ICIQ-N)
- Women of childbearing potential must have a negative urinary pregnancy test at screening (before the first study drug intake) & must agree to use a reliable method of contraception from the screening visit until 4 weeks after study drug discontinuation. Acceptable methods of birth control during participation in the study include: oral contraceptives, patch contraceptives, injection contraceptives, male condom with spermicide, diaphragm or cervical cap with spermicide, vaginal contraceptive ring, intrauterine device, surgical sterilization (bilateral tubal ligation), vasectomised partner(s) or total sexual abstinence.
- The MeNiMS co-ordinating centre will be notified if a subject becomes pregnant during the study, or changes her method of contraception

A woman is considered to be of childbearing potential unless she meets one of the following criteria: Previous bilateral salpingo-oophorectomy or hysterectomy, premature ovarian failure confirmed by a gynaecologist, aged >50 years and not treated with any kind of hormone replacement therapy for at least 2 years prior to screening, with amenorrhea for at least 24 consecutive months prior to screening, and a serum follicle stimulating hormone (FSH) level of >40 IU/L at Screening.

### Outcome measures

Primary outcome measure: Reduction in nocturia episodes per night, derived from a frequency volume chart (FVC). Analysis will be undertaken to assess the mean reduction in mean nocturia episodes. Reduced episodes of nocturia/night signifies improved sleep disturbance; 0.5/night fewer is clinically significant in older men [7]; this number may be different in MS, a condition in which there can be diverse causes of sleep disturbance.

Secondary outcome measures;

1. Improved quality of life; assessed with the MS Quality of Life Index (MSQLI) [14], a disease-specific tool to capture comprehensive quality of life dimensions, including: fatigue, urinary symptoms and general function.
2. Safety. MS disease severity at three key trial stages (before and at the end of each of the treatment periods) will be catalogued by assessing the MS-specific Expanded Disability Status Scale (EDSS) [15], the only outcome rating for MS disability accepted by Regulatory Authorities. Adverse events including MS relapses will also be documented.
3. Urinary tract symptoms; response of LUTS to melatonin treatment will be evaluated using tools of the International Consultation on Incontinence Questionnaire (ICIQ). ICIQ-MLUTS and ICIQ-FLUTS are gender specific tools for assessing severity and bother of all LUTS [16]. Nocturia-specific tools are also available within ICIQ [17]. The ICIQ-N is a short tool, which will be used in screening and at the end of the crossover washout to catalogue subjective severity. Since the ICIQ-N questions are present verbatim in ICIQ-MLUTS and FLUTS, ICIQ-N will not be co-administered with the general LUTS tools at baseline and ends of the treatment phases. ICIQ-NQoL is a nocturia-specific quality of life tool which will be used throughout the study.
4. Voided volume; the FVC will evaluate total overnight voided volume (by the standardised criteria [13]); mean voided volume will be derived by dividing total nocturnal voided volume by the number of nocturnal voids. Post void residual (PVR) will be checked during clinical assessment, with retesting during the study if clinically indicated.
5. Sleep quality; measured with the Pittsburgh Sleep Quality Index (PSQI) [18].
6. Partner’s sleep disturbance; impact of nocturia on bed-sharing partners is under-recognised. We will seek permission from study subjects to approach partners, in order to invite them to undertake qualitative interview regarding impact of nocturia on partners. This will be used to inform design of a partner-specific tool (akin to that developed for partners of men with prostate enlargement [19] for subsequent nocturia research.
7. Cognition sub-study; performance on the minimal assessment of cognitive function in MS (MACFIMS), which is an objective, internationally validated tool for specific evaluation of several key domains of cognitive function specifically affected in MS [20] and features the three key tests of the BICAMS which is undergoing international validation as the newest cognitive outcome measure for translational inquiry.

### Qualitative assessment

Qualitative Interviews will be undertaken and analysed using standard thematic methods, to capture key issues from the patient/partner perspective. Qualitative interviews will be carried out after the final treatment phase, undertaking a thematic analysis to explore issues of quality of life, mechanisms of sleep disturbance, coping strategies, impact on partners, and aspects of participating in a study. Specific aims will be to;

- Explore the patients’ perspective of nocturia.
- Explore perceptions of the intervention on nocturia and associated quality of life impact from patients’ perspective.
- Evaluate the validity of the ICIQ-NQoL in the MS population.
- Where possible partners will also be interviewed on the above perspectives to explore wider impact.

Semi structured interviews will be recorded and analysed for emerging themes, with double coding of transcripts. Sufficient interviews will be undertaken to achieve saturation of emerging themes. Qualitative interviews will be carried out after the second treatment phase has been completed. Subjects will be able to opt out of participating in the qualitative assessment; a separate consent for qualitative assessment interview will be obtained. We will ask subjects for permission to undertake qualitative assessment with partners; if allowed to approach the partner, a separate consent for qualitative assessment interview will be obtained from the partner. The study flowchart is given in (Table 1).

**Table 1:**
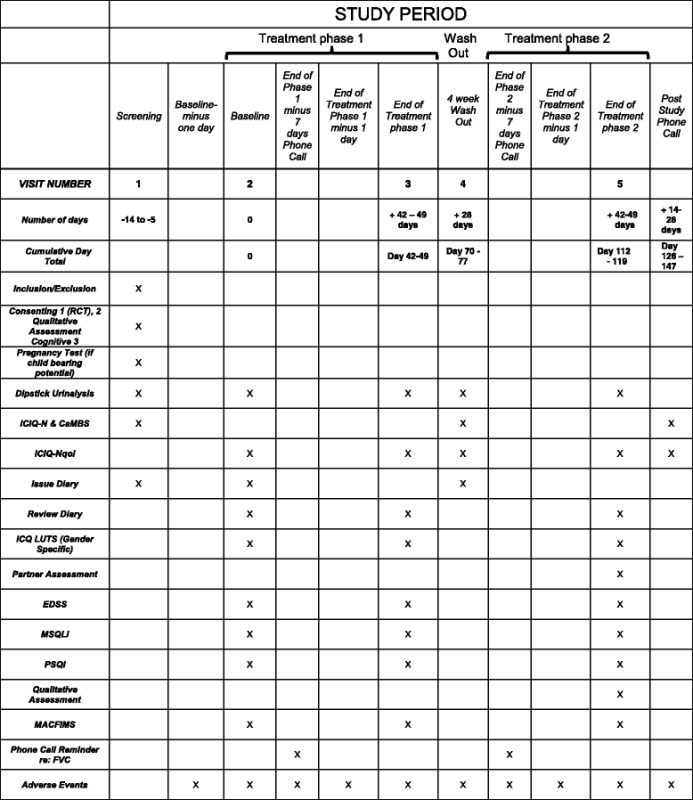
Study Flowchart

### Withdrawal criteria

A study subject will be discontinued from participation in the study if:

- Any clinically significant Adverse Event (AE) as evaluated by the PI, laboratory abnormality, intercurrent illness, or other medical condition or situation occurs such that continued participation in the study would not be in the best interest of the subject.
- The subject meets any exclusion criterion (either newly developed or not previously recognized).

Subjects are free to withdraw from participation in the study at any time upon request. Subjects who withdraw from the study will be followed up clinically as part of normal clinical care. Patients who withdraw as a consequence of Adverse Events will be followed up by the Study for 3 months and remain under clinical management thereafter if indicated. Safety data will be collected on any subject discontinued because of an AE or Serious Adverse Event (SAE) for a period of 3 months. Every effort will be made to undertake protocol-specified safety follow-up procedures under medical supervision until the symptoms of any AE resolve or the subject’s condition becomes stable. If voluntary withdrawal occurs, the subject will be asked to continue scheduled evaluations, complete an end-of-study evaluation, and be given appropriate care. All trial data to date will be held securely as per study subjects completing the protocol.

### Randomisation and enrolment procedure

Patients will be enrolled by invitation, if they fulfil all inclusion criteria and no exclusion criteria. A randomisation schedule will be generated by the website Randomization.com (http://www.randomization.com). Clinical trial materials will be packed and Qualified person (QP) certified by a contracted NHS Pharmacy, according to the randomization schedule (under Article 13 of EU directive 2001/20/EC). The medication bottles and randomization schedule will be shipped to the site pharmacy, following regulatory approvals. The randomisation schedule will be strictly controlled and remain in the pharmacy site file for emergency unblinding purposes. These features ensure the study has pharmacy controlled allocation concealment. Medication bottles will be dispensed in sequential number order, with each subject receiving Period 1 and Period 2.

Unblinding during the trial will only be undertaken in the event of a SAE or Suspected Unexpected Serious Adverse Reaction (SUSAR), or disease progression, under the authorization of the principal investigators. Emergency unblinding will be managed by North Bristol NHS Trust Pharmacy Department, according to standard operating procedure (CT/011). The study includes three MS-specific Expanded Disability Status Scale (EDSS) tests (pre-treatment and at the end of each treatment period) to monitor disease severity. Interim analysis will evaluate outcomes of the first treatment period. The analyses will provide the content of a blinded report to the Trial Management Group (TMG). If issues of safety emerge, the TMG will be empowered to request unblinded data.

### Interaction with other drugs

The subjects will not be permitted to use

- Sedative medications (CNS depressants). Taking melatonin along with sedative medications might cause excessive sleepiness. Sedative medications include clonazepam (Klonopin), lorazepam (Ativan), phenobarbital (Donnatal), zolpidem (Ambien), and others. Patients will be instructed to discuss with the trial co-ordinating office before starting such medications.

Caution will be advised for the following;

- Birth control pills (Contraceptive drugs). Taking melatonin along with birth control pills might increase the effects and side effects of melatonin. Effective methods of birth control for females of child-bearing potential are an inclusion criterion of the MeNiMS study; the trial co-ordinating office records method of contraception on the Case Report Form (CRF).
- Fluvoxamine (Luvox). Taking fluvoxamine (Luvox) can increase the amount of melatonin that the body absorbs. Taking melatonin along with fluvoxamine (Luvox) might increase the effects and side effects of melatonin. Patients will be instructed to discuss with the trial co-ordinating office before starting fluvoxamine.
- Medications for diabetes (Antidiabetes drugs). Melatonin might increase blood sugar, and thereby might decrease the effectiveness of diabetes medications. The dose of diabetes medication might need to be changed. Some medications used for diabetes include glimepiride (Amaryl), glyburide (DiaBeta, Glynase PresTab, Micronase), insulin, pioglitazone (Actos), rosiglitazone (Avandia), chlorpropamide (Diabinese), glipizide (Glucotrol), tolbutamide (Orinase), and others.
- Medications that decrease immune system function (Immunosuppressants). Melatonin might reduce the efficacy of immunosuppression. The trial co-ordinating office will discuss immunosuppression therapy with relevant patients.
- Medications that slow blood clotting (Anticoagulant/Antiplatelet drugs). Melatonin might slow blood clotting. Taking melatonin along with medications that also slow clotting might increase the chances of bruising and bleeding. Some medications that slow blood clotting include aspirin, clopidogrel (Plavix), diclofenac (Voltaren, Cataflam, others), ibuprofen (Advil, Motrin, others), naproxen (Anaprox, Naprosyn, others), dalteparin (Fragmin), enoxaparin (Lovenox), heparin, warfarin (Coumadin), and others. The trial co-ordinating office will discuss anticoagulation therapy with relevant patients.
- Nifedipine GITS (Procardia XL). Taking melatonin might decrease the effectiveness of nifedipine GITS for lowering blood pressure.
- Verapamil (Calan, Covera, Isoptin, Verelan). Verapamil (Calan, Covera, Isoptin, Verelan) can increase how quickly the body metabolises melatonin. Taking melatonin along with verapamil (Calan, Covera, Isoptin, Verelan) might decrease the effectiveness of melatonin.

### Dispensing and accountability

Dispensing, checking and collection is according to North Bristol NHS Trust Pharmacy standard operating procedures (CT/008).

Site staff will count study drug back in at return clinic visits, and remind the subject that they need to bring all their study drug and packaging back with them.

### Pharmacovigilance

Melatonin appears to cause very few side-effects in the short term, up to three months, when healthy people take it at low doses. A systematic review in 2006 looked at safety with melatonin usage for secondary sleep disturbances and sleep disorders accompanying sleep restriction [19]. The study concluded that melatonin is safe with short term use.

Unwanted effects in some people, especially at high doses (3 mg/day or more) may include: headaches, nausea, next-day grogginess or irritability, hormone fluctuations, vivid dreams or nightmares, gastrointestinal irritation, reduced blood flow and hypothermia. While no large, long-term studies that might reveal side-effects have been conducted, there do exist case reports about patients having taken melatonin for months. Melatonin can cause somnolence (drowsiness), and, therefore, caution should be shown when driving, operating machinery, etc.

#### Definitions

Adverse Event (AE): any untoward medical occurrence in a patient or clinical trial subject administered a medicinal product and which does not necessarily have a causal relationship with this treatment. An AE can therefore be any unfavourable and unintended sign (including an abnormal laboratory finding), symptom, or disease temporally associated with the use of an investigational medicinal product (IMP), whether or not considered related to the IMP.

Adverse Reaction (AR): all untoward and unintended responses to an IMP related to any dose administered. All AEs judged by either the reporting investigator or the sponsor as having reasonable causal relationship to a medicinal product qualify as adverse reactions. The expression “reasonable causal relationship” means to convey in general that there is evidence or argument to suggest a causal relationship.

Unexpected Adverse Reaction: an AR, the nature or severity of which is not consistent with the applicable product information (e.g., investigator’s brochure for an unapproved investigational product or summary of product characteristics (SmPC) for an authorised product). When the outcome of the adverse reaction is not consistent with the applicable product information this adverse reaction should be considered as unexpected. Side effects documented in the SmPC which occur in a more severe form than anticipated are also considered to be unexpected.

Serious Adverse Event (SAE) or Serious Adverse Reaction: any untoward medical occurrence or effect that;

- Results in death
- Is life-threatening – refers to an event in which the subject was at risk of death at the time of the event; it does not refer to an event which hypothetically might have caused death if it were more severe
- Requires hospitalization, or prolongation of existing inpatients’ hospitalization
- Results in persistent or significant disability or incapacity
- Results in a congenital anomaly or birth defect

Medical judgement should be exercised in deciding whether an AE/AR is serious in other situations. Important AE/ARs that are not immediately life-threatening or do not result in death or hospitalization but may jeopardise the subject or may require intervention to prevent one of the other outcomes listed in the definition above, should also be considered serious.

Suspected Unexpected Serious Adverse Reaction (SUSAR): any suspected adverse reaction related to an IMP that is both unexpected and serious.

#### Causality

Most adverse events and adverse drug reactions that occur in this trial, whether they are serious or not, will be expected treatment-related toxicities due to the drugs used in this trial. The assignment of the causality will be made by the investigator responsible for the care of the participant using the definitions in the table below. If any doubt about the causality exists the local investigator will notify the Chief Investigator.

### Relationships

**Unrelated**. There is no evidence of any causal relationship

**Unlikely**. There is little evidence to suggest there is a causal relationship (e.g. the event did not occur within a reasonable time after administration of the trial medication). There is another reasonable explanation for the event (e.g. the participant’s clinical condition, other concomitant treatment).

**Possible**. There is some evidence to suggest a causal relationship (e.g. because the event occurs within a reasonable time after administration of the trial medication). However, the influence of other factors may have contributed to the event (e.g. the participant’s clinical condition, other concomitant treatments).

**Probable**. There is evidence to suggest a causal relationship and the influence of other factors is unlikely.

**Definitely**. There is clear evidence to suggest a causal relationship and other possible contributing factors can be ruled out.

**Not assessable**. There is insufficient or incomplete evidence to make a clinical judgment of the causal relationship.

#### Reporting procedures

##### Non serious AR/AEs

All such toxicities, whether expected or not, will be recorded in the toxicity section of the relevant case report form and sent to the trial coordination centre within one month of the form being due.

##### Serious AR/AEs

Fatal or life threatening SAEs and SUSARs will be reported on the day that the local site is aware of the event. The SAE form asks for nature of event, date of onset, severity, corrective therapies given, outcome and causality (i.e. unrelated, unlikely, possible, probably, definitely). The responsible investigator will sign the causality of the event. Additional information will be sent within 5 days if the reaction has not resolved at the time of reporting.

An SAE form will be faxed to the trial coordination centre for all SAEs within 24 h. However, relapse and death due to MS, and hospitalizations for elective treatment of a pre-existing condition do not need reporting as SAEs.

In the case of suspected unexpected serious adverse reactions (SUSARs), the staff at the site will: Complete the SAE case report form & send it immediately (within 24 h, preferably by fax), signed and dated to the trial coordination centre together with relevant treatment forms and anonymised copies of all relevant investigations.

The trial coordination centre will notify the MHRA, REC and the Sponsor of all SUSARs occurring during the trial according to the following timelines; fatal and life-threatening within 7 days of notification and non-life threatening within 15 days. All investigators will be informed of all SUSARs occurring throughout the trial.

Local investigators will report any SUSARs and/or SAEs as required by their Local Research Ethics Committee and/or Research & Development Office.

Subjects will have a safety follow-up at 2 – 4 weeks by telephone call. If there has been an AE, the participant will be followed up until the AE resolves, or resolves with sequelae for a limit of 3 months and will remain under clinical management as needed.

### Loss to follow-up

Subjects lost to follow up will be contacted by telephone call and subsequently through their general practitioner. If they can be contacted, they will then be managed as per withdrawals. Records relating to all patients lost to follow up will be retained and archived according to the overall study protocol.

### Trial closure

The end of the crossover study will be defined as the point at which all study subjects have completed the data collection visits and follow up.

The trial will be closed prematurely if the TMG recommends it to the steering committee on the grounds of safety or futility. Interim analysis will be undertaken following the first treatment period. The analysis will provide the content of a blinded report to the TMG, which will consider safety/adverse effects, and assess the possibility of futility. If issues of safety emerge, the TMG will be empowered to request unblinded data. As it deems necessary, the TMG will make recommendations to the sponsor on further study conduct, including continuing, terminating, or modifying the trial. Early termination will result if clinically significant unexpected adverse effects are seen, or if the study is deemed futile, or if the beneficial effect is so obvious that curtailment can occur.

The Sponsor (Research & Innovation Department North Bristol NHS Trust) will monitor the study to ensure compliance with Good Clinical Practice procedures.

### Statistics and data analysis

Sample size is informed by (a) a previous study of melatonin, which showed a reduction in nocturia episodes of 0.5 per night to be clinically significant [7], (b) comparable crossover investigations using melatonin in other populations [21], (c) crossover trial methodology [22], and (d) project feasibility. For a two-sided test, using standard levels of statistical significance (alpha = 0.05), a sample size of *n* = 21 complete data sets would have 80% power to detect a medium to large effect size (Cohen’s d = 0.65) with 80% power, and a sample size of *n* = 34 would be needed for a medium effect size (Cohen’s d = 0.5).

The balanced two group, two period, two sequence, double-blind, randomised crossover design with wash-out period comparing treatment to control, ranks highly in the hierarchy of designs. The analyses of the resultant data under this AB/BA design may proceed using well known statistical techniques (e.g. [23]).

Primary analyses will focus on nocturia episodes per night and volume voided per night determined pre- and post-treatment for the two conditions (melatonin, placebo) within each period. The experimental structure therefore has a two by two fully repeated measures structure. The design has been structured with a large washout period and it is anticipated that there will be no carryover effects on physiological and psychological outcomes. This assumption will be tested. Period effects should also be absent and this supposition will also be tested and analysis will proceed to estimate treatment effects robust to any period effect (CROS analysis). It is recognised that outcome data may be skewed for this population and nonparametric or bootstrapped inference will be used as appropriate. Analyses will proceed on an Intent-to-Treat basis.

A missing data analysis will also be performed and sensitivity of statistical conclusions to missingness will be undertaken using standard imputation techniques. Data integrity checks will be undertaken prior to inferential analysis including a detailed graphical and summary statistics descriptive analysis. The statistician will be masked to prevent ascertainment bias. All analyses, and the reporting of analyses, will be undertaken in the spirit and guidance of the CONSORT guidelines.

### Monitoring

The TMG will consider safety/adverse effects, and assess the possibility of futility. If issues of safety emerge, the TMG will be empowered to request unblinded data. As it deems necessary, the TMG will make recommendations to the sponsor on further study conduct, including continuing, terminating, or modifying the trial. Early termination will result if unexpected adverse effects are seen, or if the study is deemed futile, or if the beneficial effect is so obvious that curtailment can occur.

#### Risk assessment

We anticipate no likely problems to result from use of a low-dose of melatonin in a study of this nature. Nonetheless, a range of purported potential effects of melatonin means the study includes three EDSS tests (pre-treatment and at the end of each treatment period) to monitor disease severity. Any clinical relapses of MS activity will also be captured as adverse events. Interim analysis will be undertaken following the first treatment period and will be submitted in a blinded report to the TMG (see above).

#### Monitoring at trial coordination centre

The trial co-ordination centre will undertake data entry checks and consent form checks, and will screen for double data entry, missing or unusual data values. Initiation and first/screening recruitment visits will be undertaken.

### Regulatory issues

Eudract Reference: 2012-004183-21 has been used for UK competent authority (MHRA) forms in order to get clinical trial authorization

Consent to enter the trial will be sought from each participant only after a full explanation has been given, an information leaflet offered and time allowed for consideration. Signed participant consent will be obtained. The right of the participant to refuse to participate without giving reasons will be respected. After the participant has entered the trial the clinician remains free to give alternative treatment to that specified in the protocol at any stage if he/she feels it is in the participant’s best interest, but the reasons for doing so will be recorded. In these cases, the subjects will remain within the trial for the purposes of follow-up and data analysis. All subjects will be free to withdraw at any time from study without giving reasons and without prejudicing further treatment.

Where possible we will gain consent from study subjects to approach partners, in order to invite them to undertake qualitative interview regarding impact of nocturia on partners and gain their consent.

#### Confidentiality

Subject’s identification data will be required for the registration process. The Trial Coordination Centre will preserve the confidentiality of subjects taking part in the trial and is registered under the Data Protection Act.

#### Access to the data

The Senior IT Manager (in collaboration with the CI) will manage access rights to the data set. Prospective new users must demonstrate compliance with legal, data protection and ethical guidelines before any data are released. We anticipate that anonymised trial data will be shared with other researchers to enable prospective meta-analyses.

Data and all appropriate documentation will be securely stored in archive for a minimum of 10 years after the completion of the trial, including the follow-up period.

#### Sponsor

North Bristol NHS Trust will act as the Sponsor for this trial. North Bristol NHS Trust holds standard NHS Hospital Indemnity and insurance cover with NHS Litigation Authority for NHS Trusts in England, which apply to this trial. The trial may be subject to inspection and audit by North Bristol NHS Trust under their remit as Sponsor, the Trial Coordination Centre and other regulatory bodies to ensure adherence to GCP.

### Data Monitoring Committee

We aim to establish an independent Data Monitoring Committee (DMC) comprising an independent chair and two other independent members, to monitor accumulating data during the course of the trial. All personnel will have undertaken the mandatory Good Clinical Practice (GCP) training.

### Trial management

A Trial Management Group (TMG) has been appointed and will be responsible for overseeing the progress of the trial. The day-to-day management of the trial will be co-ordinated through the MeNiMS Trial Coordination Centre. All publications and presentations relating to the trial will be authorised by the Trial Management Group.

### Publication policy

The main forms of dissemination will be through the academic press and by lay summaries on websites and other more accessible forms for patients. All participants will be offered a lay summary of the main findings of the study. This will be adapted for dissemination through public channels. Dissemination to clinicians will be through papers in major urology and neurology journals and conferences, workshops and presentations to national meetings.

## Discussion

This study will evaluate whether melatonin reduces the frequency of nocturia episodes in MS patients, and therefore whether ‘Circadin’ has the potential to reduce LUTS and fatigue, and improve cognition and overall quality of life. Nocturia and sleep disturbance are prevalent and bothersome aspects of MS, and a safe and effective therapy would be a valuable development. Melatonin may improve either or both aspects, and has a good safety profile. It is unlikely to affect necessary physiological function of the lower urinary tract or the gut. Thus, it does not carry the same concerns about side effects of drugs used to treat overactive bladder syndrome, such as constipation or impaired bladder emptying.

The trial includes a wide range of outcome measures, since the effect on nocturia and sleep quality may lead to additional effects on cognitive function. Since this is a clinical trial of an investigational medical product (using an established drug but outside its licensed indication), the measures also emphasises safety assessments, including detailed assessment of MS severity.

## Abbreviations

CaMBS: Cambridge Multiple Sclerosis Basic Score
EDSS: Expanded Disability Status Scale
F-LUTS: Female Lower Urinary Tract Symptoms
FVC: Frequency volume chart
ICIQ: International Consultation on Incontinence Questionnaires
ICIQ-FLUTS: Female- specific tool for assessing severity and bother of all LUTS
ICIQ-MLUTS: Male-specific tool for assessing severity and bother of all LUTS
ICIQ-N: International Consultation on Incontinence-Nocturia
ICIQ-NQoL: A nocturia-specific quality of life tool which will be used throughout the study
LUTS: Lower urinary tract symptoms
MACFIMS: Minimal assessment of cognitive function in MS
MeNiMS: Study Acronym: Melatonin for Nocturia in Multiple Sclerosis
M-LUTS: Male lower urinary tract symptoms
MS: Multiple sclerosis
MSQLI: Multiple sclerosis quality of life index
PSQI: Pittsburgh Sleep Quality Index
